# M2 Polarization of Monocytes-Macrophages Is a Hallmark of Indian Post Kala-Azar Dermal Leishmaniasis

**DOI:** 10.1371/journal.pntd.0004145

**Published:** 2015-10-23

**Authors:** Debanjan Mukhopadhyay, Shibabrata Mukherjee, Susmita Roy, Jane E. Dalton, Sunanda Kundu, Avijit Sarkar, Nilay K. Das, Paul M. Kaye, Mitali Chatterjee

**Affiliations:** 1 Department of Pharmacology, Institute of Postgraduate Medical Education and Research, Kolkata, West Bengal, India; 2 Centre for Immunology and Infection, Hull York Medical School and Department of Biology, University of York, York, United Kingdom; 3 Department of Dermatology, Calcutta Medical College, Kolkata, West Bengal, India; Yale School of Public Health, UNITED STATES

## Abstract

The high level of functional diversity and plasticity in monocytes/macrophages has been defined within *in vitro* systems as M1 (classically activated), M2 (alternatively activated) and deactivated macrophages, of which the latter two subtypes are associated with suppression of cell mediated immunity, that confers susceptibility to intracellular infection. Although the *Leishmania* parasite modulates macrophage functions to ensure its survival, what remains an unanswered yet pertinent question is whether these macrophages are deactivated or alternatively activated. This study aimed to characterize the functional plasticity and polarization of monocytes/macrophages and delineate their importance in the immunopathogenesis of Post kala-azar dermal leishmaniasis (PKDL), a chronic dermatosis of human leishmaniasis. Monocytes from PKDL patients showed a decreased expression of TLR-2/4, along with an attenuated generation of reactive oxidative/nitrosative species. At disease presentation, an increased mRNA expression of classical M2 markers *CD206*, *ARG1* and *PPARG* in monocytes and lesional macrophages indicated M2 polarization of macrophages which was corroborated by increased expression of CD206 and arginase-1. Furthermore, altered vitamin D signaling was a key feature in PKDL, as disease presentation was associated with raised plasma levels of monohydroxylated vitamin D_3_ and vitamin D3- associated genes, features of M2 polarization. Taken together, in PKDL, monocyte/macrophage subsets appear to be alternatively activated, a phenotype that might sustain disease chronicity. Importantly, repolarization of these monocytes to M1 by antileishmanial drugs suggests that switching from M2 to M1 phenotype might represent a therapeutic opportunity, worthy of future pharmacological consideration.

## Author Summary

Monocyte/macrophage subsets following their polarization by the microenvironement serve as important immune sentinels that play a vital role in host defense and homeostasis. The polarization of macrophage function has been broadly classified as M1 (classical) and M2 (alternate) activation, wherein M1 polarised cells display a strong pro-inflammatory microbicidal response, while M2 polarization is linked to production of an anti-inflammatory milieu leading to tissue regeneration and wound healing. Data pertaining to macrophage polarization are primarily derived from murine models, but increasing evidence is highlighting the inadequacy of direct inter-species translation. In leishmaniasis, a protozoan infection caused by the genus *Leishmania*, manipulation of host macrophage function is central to pathogenesis. In this study we report that monocyte/macrophage subsets in Post kala-azar dermal leishmaniasis are polarized to an M2 phenotype. This study provides insights into systemic and local regulation of macrophage/ monocyte functions in this important human disease and highlights the influence of immunomodulatory anti-leishmanial chemotherapy on macrophage/monocyte polarization.

## Introduction

Leishmaniases comprise a group of heterogeneous parasitic diseases caused by the protozoan parasite *Leishmania* with its spectrum ranging from a self-healing cutaneous variant to the often fatal visceral leishmaniasis (VL). Post kala-azar dermal leishmaniasis (PKDL) is the dermal sequel of VL, wherein *Leishmania* parasites remain restricted to the skin and manifest as nodular, papular, hypopigmented macular lesions, erythematous plaques and/or a mixed phenotype, termed as polymorphic [1]. The etiopathogenesis of PKDL is still unclear and a consensus is yet to emerge regarding possible causes for the generally viscerotropic *L*. *donovani* parasite to generate PKDL. In PKDL, similar to other leishmaniasis, *Leishmania* have developed several strategies to outmanoeuvre host immunity via subverting and/or suppressing macrophage microbicidal activities [2].

It is universally accepted that monocytes-macrophages have a range of biological roles being inducers, regulators and effectors of innate and acquired immunity. They also actively participate in physiological processes associated with wound healing and tissue repair [3]. Upon stimulation with Th1-associated cytokines, notably IFNγ, they acquire a heightened effector function against intracellular pathogens, referred to as a classically activated or M1 phenotype. Conversely, in the milieu of Th2 associated cytokines e.g. IL-4, IL-13, IL-33, TGF-β and IL-10 [4] or via microbial triggers [5], there is M2 polarization or alternative activation. The differentiation of M1 and M2 monocytes/macrophages is regulated by cardinal genes that include inducible nitric oxide synthase (*iNOS*), arginase 1 (*ARG1*), mannose receptor (*CD206*) and *Fizz1* among others [4, 6]. M2 macrophages can impede protective immunity to protozoan infection. In an animal model of cutaneous leishmaniasis (CL), Holscher et al., [7] demonstrated that alternative activation favoured disease progression, whereas the impairment of M2 macrophages significantly delayed disease progression. In studies regarding human leishmaniasis, raised levels of arginase have been demonstrated in neutrophils and low density granulocytes [8,9]. Similarly, in diffuse cutaneous leishmaniasis (DCL), a more chronic form of leishmaniasis more akin to PKDL, there was an elevation of the arginase pathway (arginase-1, ornithine decarboxylase and polyamine) [10]. Furthermore, this study validated that inhibition of arginase-1 or ornithine decarboxylase abrogated parasite replication within human macrophages [10].

Our understanding of the phenotypic and functional complexity of M2 monocytes-macrophages is limited by discordance between data derived from murine vs. human systems [11]. Unlike classically activated macrophages, where human and murine cells respond similarly, the molecular phenotype of alternatively activated macrophages in mice and humans have to date shown a limited overlap [12]. Additionally, as the dichotomy of M1 vs. M2 is still not clearly defined [4], it emphasized the importance of undertaking human studies, especially with regard to infectious diseases. Accordingly, this study aimed to delineate in patients with PKDL the activation status of monocytes in peripheral blood and dermal macrophages, thus providing the first characterization of M2 polarized macrophages in human dermal leishmaniasis. Furthermore, we demonstrate the repolarization of monocytes after antileishmanial chemotherapy suggesting that therapeutic options designed to restore the M1:M2 balance may be effective in disease elimination.

## Materials and Methods

### Ethics statement

The study received approval from School of Tropical Medicine Kolkata (STM) and Institute of Postgraduate Medical Education and Research, Kolkata. Studies on tissue biopsies were approved by the UK National Research Ethics Service. Written informed consent was obtained and for a minor, their legally accepted representative provided the same.

### Study population

**Fig 1:**
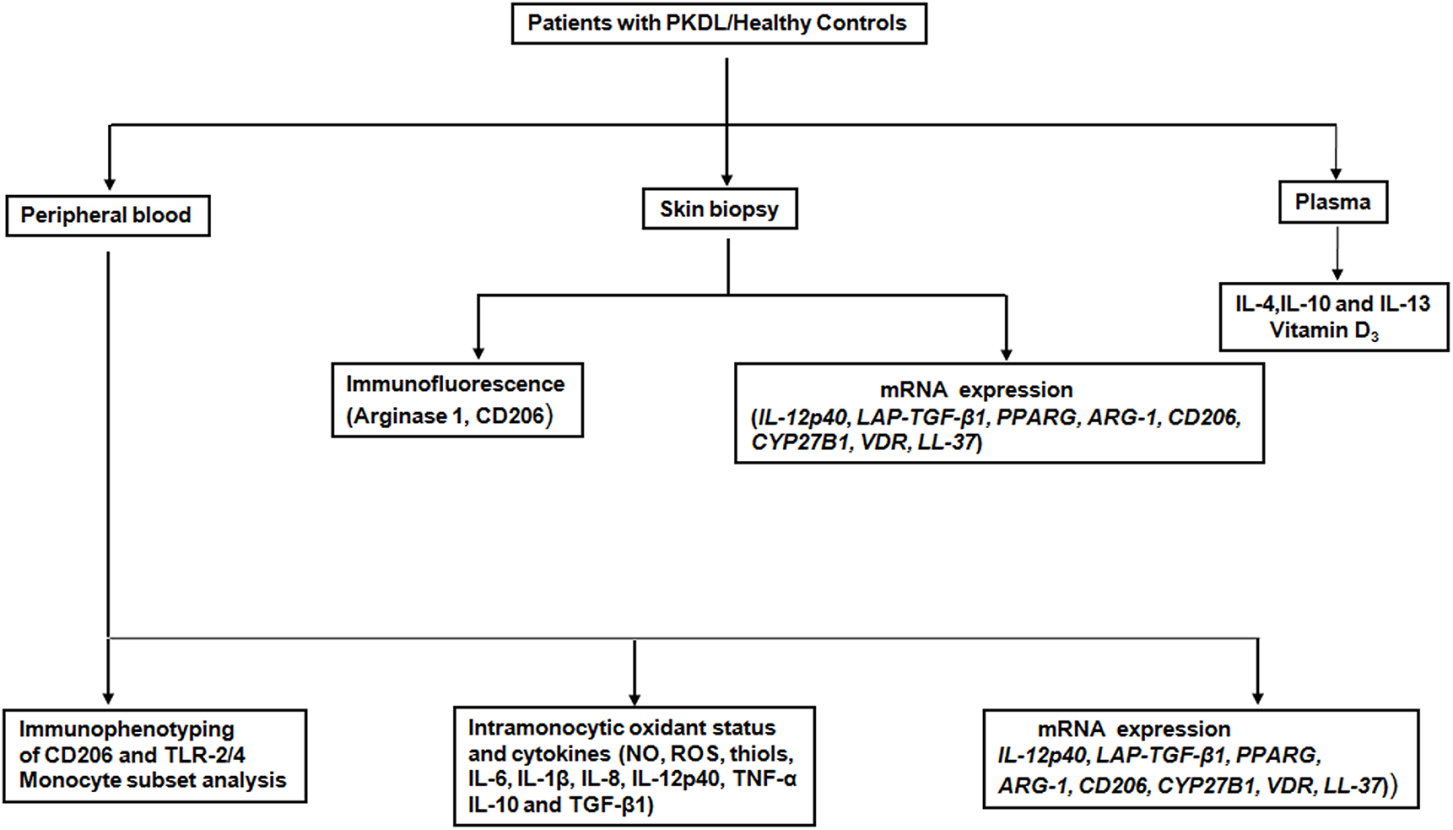
Immunological analysis of patients with PKDL. Schematic analyses performed on patient groups and healthy controls.

From 2010–11, patients suspected with PKDL (n = 34; **Table 1**) were recruited from the Dermatology Outpatient Department, STM, based on clinical features and a prior history of VL or were resident in a VL endemic area. Diagnosis was confirmed by the rK39 strip test (In Bios International, Seattle, USA) and ITS-1 PCR from skin biopsies. Patients received Miltefosine (100 mg/day p.o. 4 months, n = 14) or sodium antimony gluconate (20 mg/kg b.w., i.m. 4 months, n = 20). Age and sex-matched healthy volunteers (n = 15) recruited from non-endemic areas were seronegative for anti-leishmanial antibodies. Samples were collected at disease presentation and upon completion of treatment (Table 1), which varied from 3–4 months, based on patient compliance. The analysis plan is shown in **Fig 1**.

**Table 1:** Study Population.

Patient Characteristics	PKDL (n = 34)	Healthy controls (n = 15)
Age (years) range (Mean ± SEM)	9–80 (32.94 ± 2.76)	23–33 (27.70 ± 92)
Sex (Male/Female)	7.5:1	2:1
Cases reporting history of VL	26	None
History of VL, range in years (Mean ± SEM)	0.5–42 (6.97± 1.90)	N.A
Duration of disease, range in years (Mean ± SEM)	0.3–20 (5.30 ± 0.87)	N.A
Lesional profile		
(i) Polymorphic	30	N.A.
(ii) Macular	4	N.A.
Hemoglobin (g/dl)	11.96 ± 0.89	12.82 ± 0.57
Total leukocyte count (cells/mm^3^)	6836 ± 552.20	7560 ± 535.40
Differential leukocyte count (%)		
(i) Neutrophils	60.6 ± 1.9	68.2 ± 2.2
(ii) Lymphocytes	32.1 ± 1.5	28.2 ± 2.4
(iii) Monocytes	2.1 ± 0.5	1.4 ± 0.2
(iv) Eosinophils	4.9 ± 0.7*	2.2 ± 0.4
(v) Basophils	0.8 ± 0.5	0.00

Values are mean ± SEM.

N.A = Not Applicable

*indicates p<0.05 than healthy controls

### Isolation of monocytes

Peripheral blood diluted with phosphate buffered saline (0.01M pH 7.2, PBS) was layered over a monocyte isolation medium (3:1; HiSep LSM-1073) and centrifuged (400*g*, 30 minutes). The monocyte rich interface was washed twice in PBS and then resuspended in RPMI-1640, supplemented with penicillin (100 U/mL), streptomycin (100 μg/mL) and 10% heat-inactivated fetal bovine serum. To confirm purity, monocytes were initially gated on their forward vs. side scatter characteristics followed by CD14 positivity. The absence of PMNs was checked by CD15 negativity and cells were used for immunophenotyping and/or mRNA expression studies.

### Immunophenotyping for Toll like receptors (TLRs)

After isolation of monocytes, they were surface stained with anti human CD14 FITC (Biolegend, San Diego, CA, USA) and incubated for 30 minutes at room temperature (RT). The cells were then washed twice with PBS followed by fixation and permeabilization by incubating with a fix-perm buffer (2% paraformaldehyde + 0.05% saponin + 3% FBS in PBS) for 20 minutes at RT. Cells were then stained with anti human TLR-2 PE and TLR-4 FITC (BD Biosciences, San Jose, CA, USA) for 15 minutes. Cells were then washed twice and resuspended in PBS-2% FBS for acquisition in a flow cytometer (BD FACS Calibur, BD Biosciences, San Jose, CA, USA).

### Estimation of plasma cytokines

Plasma levels of circulating cytokines, IL-4, IL-10 and IL-13 were measured in patients with PKDL and healthy controls by sandwich ELISA according to the manufacturer’s instructions (Immunotools, Friesoythe, Germany).

### Immunophenotyping of monocytes

Whole blood (100 μL) was stained for 20 minutes with either CD14 FITC and CD16 PE or antihuman CD14-PerCP and CD206-Alexafluor 488 with appropriate isotype controls [Biolegend, San Diego, USA, 13]. Cells were then washed twice with PBS, resuspended in 400 μL of PBS and acquired in a Flow Cytometer.

### Intracellular staining of monocytes

For intracellular staining, monocytes (1x10^6^ cells/well/mL) were cultured overnight, followed by Brefeldin A (1 μg/mL, 4h) and surface stained with CD14-FITC [Biolegend, San Diego, USA. They were then stained for IL-6-PE, IL-1β-PE (eBioscience, San Diego, USA)], IL-8-APC, IL-12p40-PE, Latency associated peptide (LAP)-TGF-β1-APC (Biolegend, San Diego, USA) along with isotype controls and acquired in a flow cytometer.

5000 monocytes were acquired and data analyzed by Cell-Quest pro software. The frequency of cells with a particular phenotype was expressed as % of monocytes and was calculated by dividing percentages of the upper right quadrant (CD14^+^ marker^+^) by the sum of the upper and lower right quadrant (CD14^+^ marker^-^).

### Measurement of the oxidant status

Monocytes (5x10^5^) after centrifugation (400*g*, 5 minutes) were resuspended in PBS and stained with 4,5-diaminofluorescein diacetate (DAF-2DA, 2 μM, 30 minutes,37°C, Cayman Chemicals, Ann Arbor, Michigan, USA). The fluorescence of DAF-2T was acquired in a flow cytometer in the FL-1 channel [14].

The generation of ROS and levels of non protein thiols was measured in monocytes (5 x 10^5^/mL) stained with 5-(and-6)-carboxy-2',7'-dichloro dihydrofluorescein diacetate, acetyl ester (CMH_2_DCFDA, 2.5 μM) and 5-chloromethylfluorescein diacetate (CMFDA, Molecular Probes, Carlsbad, CA, USA) respectively [15]; the fluorescence of DCF and CMF was acquired in a flow cytometer. Superoxide production was measured using the cytochrome c reduction assay [16].

For analysis, monocytes were gated on their forward vs. side scatter characteristics as previously shown [14]; 5000 monocytes were acquired and data analyzed by Cell-Quest pro software. The expression of markers was indicated as geometric mean fluorescence channel or GMFC. To minimise day to day experimental variation and auto-fluorescence, an unstained control was included for each sample. Patient samples were analyzed alongside a healthy control, to minimise the effects of any temporal changes in experimental setup

### Isolation of RNA and reverse transcriptase-PCR from peripheral blood mononuclear cells

Using total RNA extracted from monocyte enriched PBMCs (1x10^6^ cells, Ambion, Life Technologies, Carlsbad, CA, USA), reverse transcriptase-PCR was performed on RNA (50 ng) with a one-step reverse transcriptase-PCR kit (Qiagen, Hilden, Germany) using gene-specific primers for *IL-12p40*, *ARG1*, *CD206*, Peroxisome proliferator activated receptor gamma *(PPARG*), Vitamin D receptor (*VDR*), 25-Hydroxyvitamin D_3_ 1-alpha-hydroxylase (*CYP27B1*), *LL-37* (cathelicidin) and *β-actin* (S1 Table). Primers were designed using NCBI gene bank reference sequences of human genes and their specificity for humans were confirmed by Basic Local Alignment Test (BLAST) in NCBI. The amplification cycle comprised 35 cycles of denaturing (94°C for 30 seconds), annealing for 30 seconds (varying temperature for each primer set; S1 Table), extension (72°C for 60 seconds), and a final extension at 72°C (10 minutes). Products were resolved on agarose gels (2%) containing ethidium bromide (0.5 mg/mL), observed and analyzed in G-BOX gel doc [Syngene, Cambridge, UK] using Gene Tools (Version 4.01.04) software. The values were normalized to β-actin, which was considered as 100% for each individual.

### Detection of Arginase-1 and Mannose receptor

Immunofluorescent staining was performed on paraffin embedded skin biopsies, mounted on glass slides, deparaffinised in xylene and then rehydrated in graded alcohol. For antigen retrieval, slides were placed in a pre-warmed antigen retrieval solution (S1699 DAKO citrate buffer pH 6.0, diluted 1:10, Cambridge, UK) and incubated in a water bath for 30 minutes at 95°C. The slides were then brought to room temperature (20 minutes) and washed with PBS. After blocking the non specific binding sites with PBS + 5% goat serum for 30 minutes, they were stained overnight at 4°C with anti human CD68 (Abcam, Cambridge, UK), 1: 500 dilution in PBS and/or rabbit anti human arginase-1 (Protein tech, Manchester, UK), 1: 50 in PBS, rabbit anti human CD206 (Protein tech, Manchester, UK), 1:100 in PBS along with appropriate isotype matched controls. After three washings with PBS + 0.05% BSA, binding was detected using secondary antibodies, anti mouse Alexa fluor 594 and anti mouse Alexa fluor 488 (diluted 1:200 in PBS for CD68 Invitrogen, Life Technologies Ltd., Paisley, UK), anti rabbit Alexa fluor 488 (diluted 1:200 in PBS for arginase-1, Invitrogen, Life Technologies Ltd., Paisley, UK) and anti rabbit Alexa fluor 647 (diluted 1:200 in PBS for CD206, Invitrogen, Life Technologies Ltd., Paisley, UK). All incubations were for 1 h at room temperature in the dark and followed by three washings. The slides were then incubated with DAPI (1 μg/mL, 200 μL, 10 minutes) and finally mounted overnight at 4°C with Pro-long Gold anti-fade (Invitrogen, Life Technologies Ltd., Paisley, UK). The images were captured in an inverted LSM 710 Confocal microscope (Carl Zeiss Microimaging, Cambridge, UK) and analyzed via LSM 7500 software and image J software.

### Measurement of plasma 25(OH) Vitamin D_3_

Plasma levels of 25(OH) Vitamin D_3_ were measured using a 25 hydroxyvitamin D radio immunoassay kit (DiaSorin, Stillwater, Minnesota, USA), range being 9.0–37.6 ng/mL and sensitivity was 1.5 ng/mL.

### Statistical analysis

Data was analyzed between groups by Kruskal-Wallis test followed by Dunn’s multiple comparison tests for non-parametric data for analysis of variance. For non-parametric paired data, Wilcoxon signed rank test was performed using Graph Pad Prism software (version 5.0), p<0.05 being significant. All data are expressed as mean ± SEM and horizontal bars in graphs indicate SEM.

## Results

### Study population

Patients with PKDL (n = 34; Table 1) showed a male preponderance, the male: female ratio being 7.5:1. Majority had polymorphic lesions i.e. hypopigmented macules and nodules/papules, while a minority showed exclusively hypopigmented macules. The rk39 test was positive in 33/34 (the rk39 negative patient was confirmed by ITS-1 PCR) and in polymorphic lesions, the presence of Leishman Donovan bodies was identified by Giemsa staining. Irrespective of treatment, the assessment of cure was clinical and parasitological (ITS-1 PCR negative). At presentation, their hemoglobin levels and leukocyte counts were comparable with controls i.e. no anemia or pancytopenia which are consistent features of VL. In patients, a maximum of 10 ml blood was provided which yielded 1–2 x 10^7^ cells. As 5 x 10^5^–2 x 10^6^ cells were required per analysis, all markers could not be evaluated in each patient, and patients were randomly selected for individual assays.

### Monocytes have a reduced expression of TLR2 and TLR4

**Fig 2:**
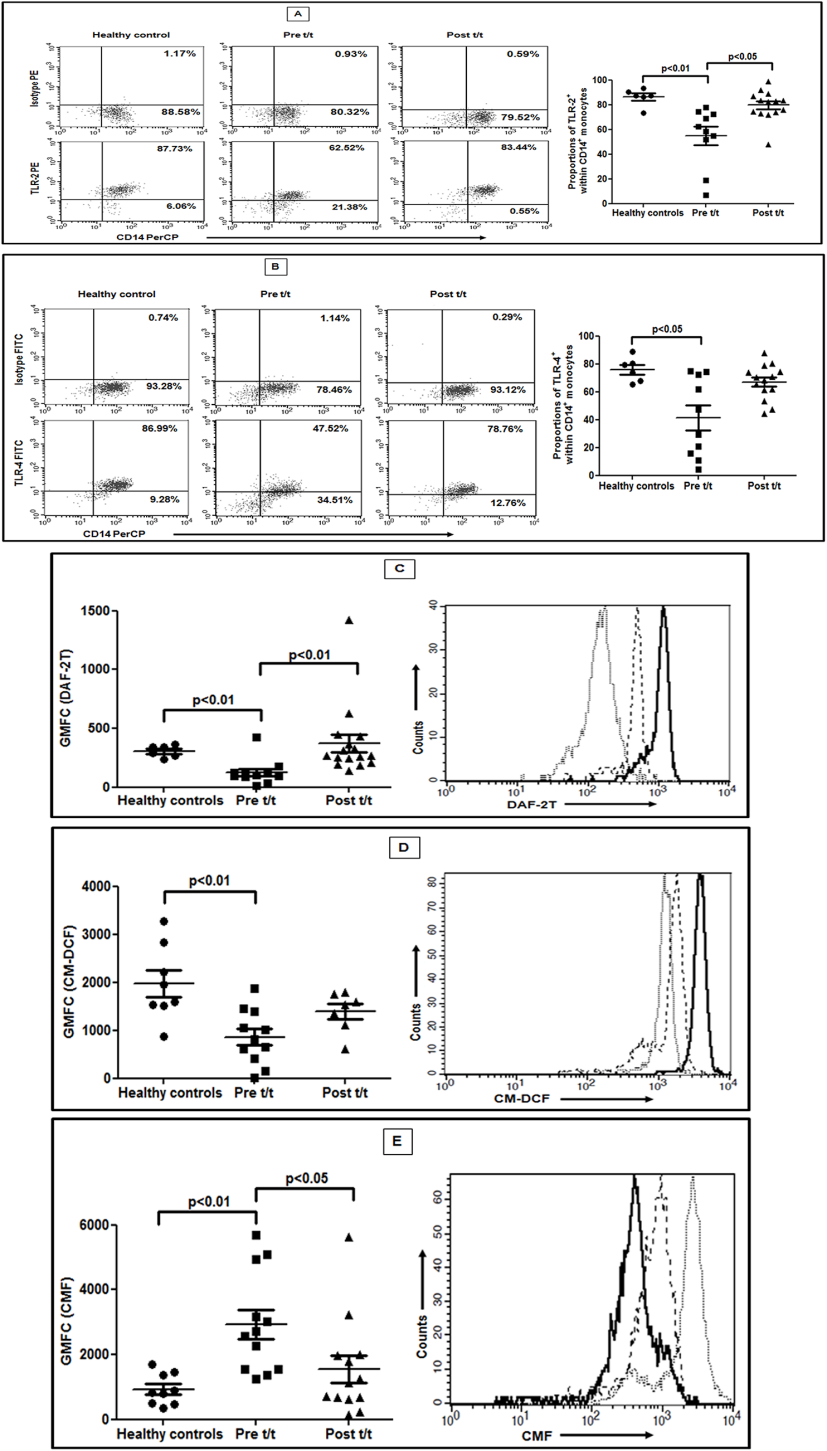
Decreased expression of TLR-2 and -4 and altered redox status within monocytes. **A-B**: Representative data showing expression of TLR-2 (**A**) and TLR-4 (**B**), within CD14^+^ monocytes in a healthy control, a patient with PKDL (Pre t/t) and post treatment (Post t/t). Isotype control staining is also shown. *Scatter plots showing frequency of CD14^+^ monocytes expressing TLR-2 or TLR-4 in healthy controls (●), patients with PKDL (Pre t/t, ■) and post treatment (Post t/t, ▲). *The proportion of CD14^+^TLR-2^+^ and CD14^+^TLR-4^+^ monocytes was calculated by dividing the percentages of upper right quadrant with the sum of upper and lower right quadrant. **C:** Scatter plots showing intracellular NO in circulating monocytes of healthy controls (●), patients with PKDL (Pre t/t, ■) and post treatment (Post t/t, ▲). Representative histogram overlay showing DAF-2T fluorescence in monocytes from a healthy control (**—**), patient with PKDL (…) and on completion of treatment (**—-**). **D:** Scatter plots of intramonocytic generation of ROS as for C above. A representative histogram overlay showing CM-DCF fluorescence, as for D above. **E:** Scatter plots indicating levels of intracellular non-protein thiols as for C above. A representative histogram overlay showing CMF fluorescence as for D above. For analysis, monocytes were initially gated on their forward vs. side scatter characteristics followed by fluorescence of CD14.

In PKDL, as compared to healthy controls, the frequency of CD14^+^ monocytes expressing TLR-2^+^ was significantly reduced (54.70 ± 7.52% vs. 86.32 ± 2.78%; p<0.01; Fig 2A), as was the frequency of TLR-4^+^ monocytes (41.26 ± 8.90% vs. 75.95 ± 3.56%; p<0.05; Fig 2B). Treatment significantly increased TLR-2^+^ monocytes (79.67 ± 3.23, p<0.05, Fig 2A) and became comparable with healthy controls. A similar scenario was demonstrated with TLR-4^+^ (66.07 ± 3.21; Fig 2B). In terms of expression (GMFC), TLR-2 was significantly downregulated in patients vs. healthy controls (7.30 ± 1.56 vs. 29.44 ± 4.03) and reverted post-treatment (30.52 ± 4.88, p<0.05). However, the expression of TLR-4 was comparable during active disease with healthy controls (16.33 ± 3.59 vs. 9.01 ± 1.24) and post treatment (10.24 ± 0.84).

### Monocytes demonstrated an impaired oxidative burst

At presentation, the *ex-vivo* levels of NO in monocytes was significantly diminished as compared to controls (GMFC, 125.40 ± 32.74 vs. 306.30 ± 20.78 respectively; p<0.01; Fig 2C). With treatment, monocytes regained their ability to generate NO (371.50 ± 76.51; p<0.01 vs. pre-treatment; Fig 2C). Similarly, the generation of ROS was significantly attenuated at presentation *vis-a-vis* controls (858.70 ± 171.70 vs. 2132.00 ± 259.90 respectively; p<0.01; Fig 2D). Treatment increased fluorescence, but remained lower than controls (1396.00 ± 158.20; Fig 2D). Alongside, the production of superoxide too was significantly lowered at disease presentation (1.98 ± 0.39 vs. 4.40 ± 0.12 nM, p<0.01) but changed minimally with treatment (3.00 ± 0.79 nM).

Variations in the anti-oxidant status impact on the redox balance and thereby on macrophage host defence functions. The intramonocytic levels of non protein thiols which primarily comprise glutathione were examined in terms of CMF derived fluorescence, wherein increased fluorescence indicated enhanced intracellular levels of non protein thiols [15]. At disease presentation, the fluorescence of CMF was significantly higher than controls (2931.00 ± 445.20 vs. 926.00 ± 160.60; p<0.01; Fig 2E). Importantly, it negatively correlated with decreased levels of ROS (r = -0.57), corroborating the presence of a robust anti-inflammatory milieu. Treatment significantly attenuated fluorescence *vis a vis* active disease (1541.00 ± 415.90, p<0.05, Fig 2E).

Monocytes can differentiate into inflammatory or anti-inflammatory subsets, but their classification in relation to functional phenotypes has not been precisely defined. Three subsets of blood monocytes, namely classical (CD14^++^CD16^−^), intermediate (CD14^++^CD16^+^), and non-classical (CD14^+^CD16^++^) have been described and attributed with discrete functions [17]. At disease presentation, there was a minimal decrease in the proportion of classical (CD14^++^CD16^-^) monocytes as compared to healthy controls (81.20 ± 4.83% vs. 89.60 ± 1.20%, S1 Fig). There was a nominal increase in the intermediate variant (CD14^++^CD16^+^) being 5.46 ± 1.19% vs. 3.46 ± 0.79%, S1 Fig) and the non-classical phenotype (CD14^+^CD16^++^; 14.29 ± 3.46% vs. 6.90 ± 1.56%, S1 Fig). With treatment, the frequency of classical (86.20 ± 2.19%, S1 Fig), intermediate (3.91 ± 1.28%, S1 Fig) or non-classical monocytes (9.74 ± 2.06%, S1 Fig) was comparable with healthy controls.

### PKDL patients showed features of M2 polarization in peripheral blood

**Fig 3:**
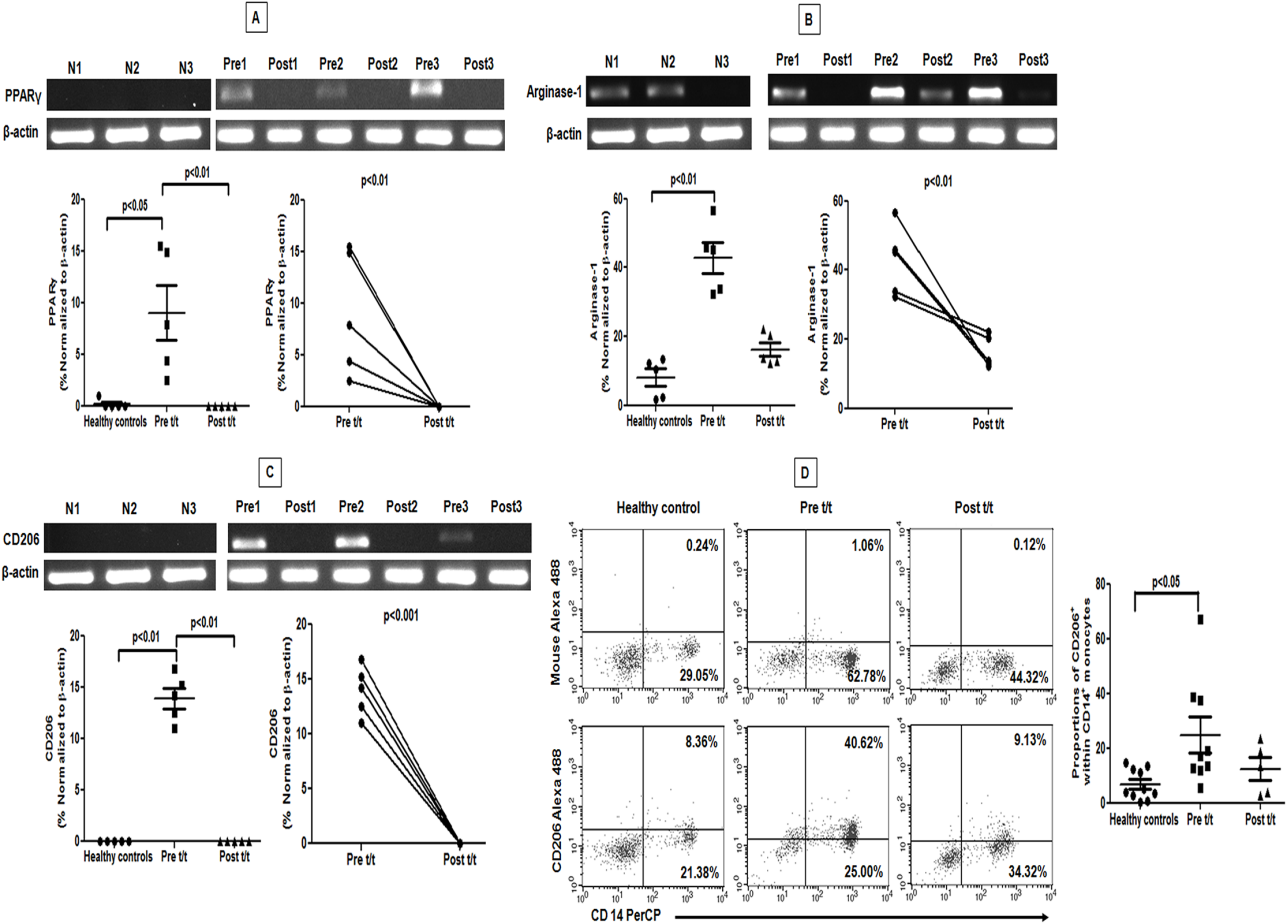
Raised expression of PPAR-γ was accompanied by an increased expression of arginase-1 and mannose receptor in circulating monocytes. **A-C:** Representative mRNA expression in circulating monocytes of PPAR-γ (A), arginase-1 (B) and mannose receptor (C) sourced from healthy controls (N 1–3), patients with PKDL (Pre 1–3) and post treatment (Post 1–3). Scatter plots indicate RT-PCR products in healthy controls (●), patients with PKDL (Pre t/t, ■) and after treatment (Post t/t, ▲). These RT-PCR products were quantified by densitometric analysis after normalization with β-actin, along with before and after plots of the same. **D:** Representative quadrant plot showing frequency of CD206 in a healthy control, a patient with PKDL (Pre t/t) and post treatment (Post t/t). Scatter plots shows frequency of CD206 within CD14 gated monocytes of healthy controls (●), patients with PKDL (Pre t/t, ■) and after treatment (Post t/t, ▲). The proportion of CD14^+^CD206^+^ monocytes was calculated by dividing the percentages of upper right quadrant with the sum of upper and lower right quadrant.

As decreased generation of reactive oxygen and nitrogen radicals are suggestive of an alternative activation [4, 18], monocytes from PKDL patients were examined for a M2 phenotype. In circulating monocytes from controls and treated patients, the mRNA expression of nuclear receptor *PPARG* which regulates oxidative metabolism in macrophages [19, 20] was minimal, but increased ~50-fold during active disease (Fig 3A). The arginase activity, being downstream of PPARγ signaling [4, 20] was also enhanced in PKDL [13]. It was supported by a 5.29 fold increase in mRNA accumulation of *ARG1* in circulating monocytes (p<0.01; Fig 3B), which with treatment significantly decreased by 2.6 fold (p<0.01; Fig 3B). In circulating monocytes from controls, mRNA accumulation of mannose receptor (*CD206/MR*) was negligible (Fig 3C), but increased 14 fold at disease presentation, and returned to baseline after treatment (p<0.01, Fig 3C). This translated into an enhanced surface expression of CD206 on monocytes during active disease and decreased with treatment (p<0.05, Fig 3D). Collectively, blood monocytes from PKDL patients showed strong evidence of M2 polarization, which with disease resolution repolarized to a M1 phenotype.

### Altered intramonocytic cytokine production

**Fig 4:**
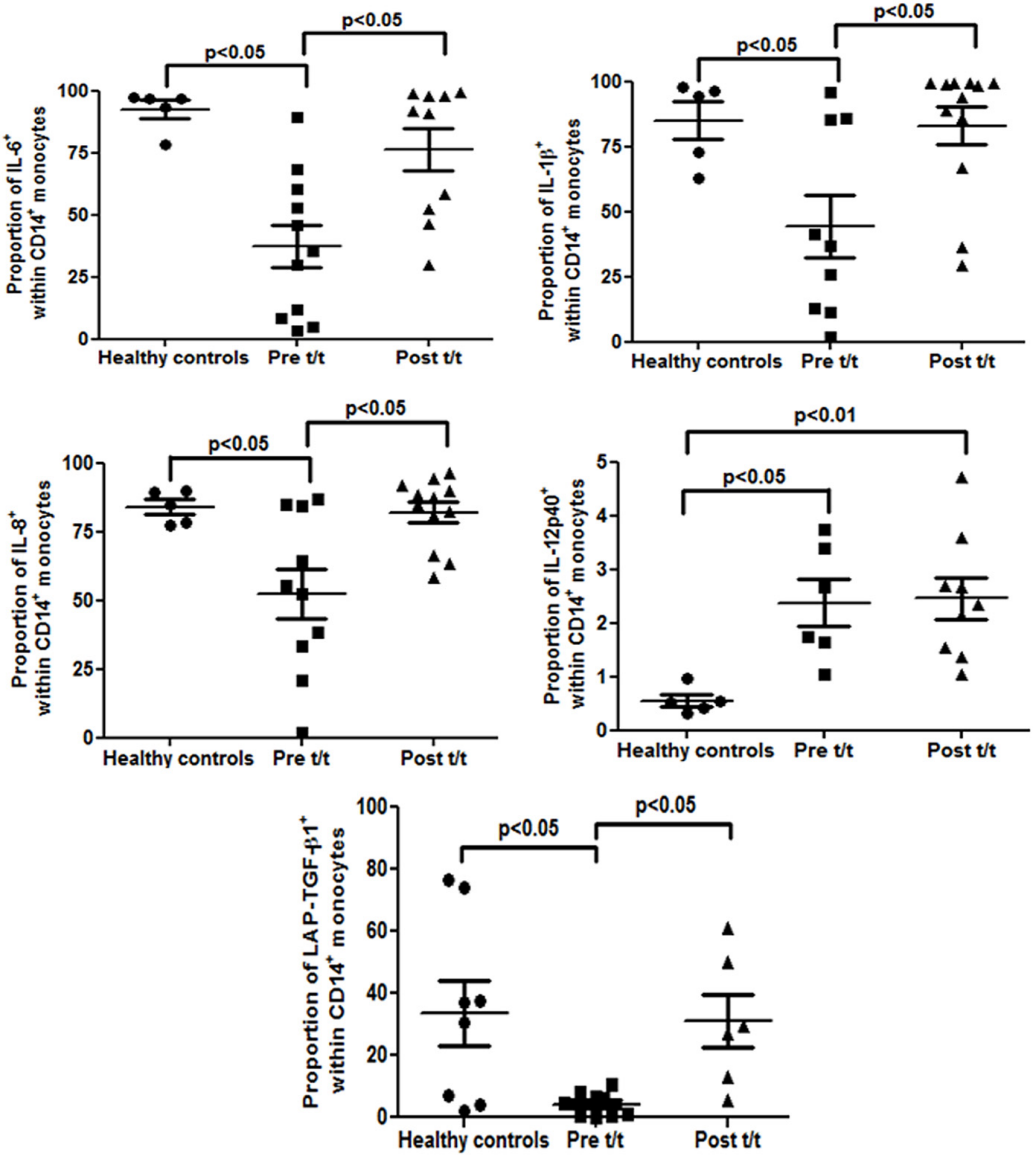
Analysis of intramonocytic status of cytokines in PKDL. Scatter plots indicating the % of CD14^+^ monocytes expressing the cytokine in healthy controls (●), patients with PKDL (Pre t/t, ■) and after treatment (Post t/t, ▲). The proportion of CD14^+^IL-6^+^, CD14^+^IL-1β ^+^, CD14^+^IL-8^+^, CD14^+^IL-12p40^+^, CD14^+^LAP-TGF-β1^+^monocytes was calculated by dividing percentages of the upper right quadrant with sum of the upper and lower right quadrant (as shown in S2 Fig).

In PKDL, a mixed cytokine profile with a Th2 bias has been reported [13, 21], but the contribution of monocytes remains unclear. We analyzed the intracellular cytokine producing ability of CD14^+^ monocytes by gating monocytes initially on their morphology, followed by CD14 expression and then calculated the % of CD14^+^-cytokine positive cells. At disease presentation, the frequency of CD14^+^IL-6^+^ monocytes was significantly attenuated *vis a vis* controls (37.31 ± 8.67% vs. 92.56 ± 3.59%, p<0.05, Fig 4 and S2A Fig), but increased significantly post-treatment (76.36 ± 8.42%, p<0.05, Fig 4 and S2A Fig). Similarly, IL-1β showed a significant 1.92 fold decrease *vis-a-vis* controls (44.17 ± 11.99% vs. 84.88 ± 7.22%, p<0.01, Fig 4 and S2B Fig) and reverted with treatment (82.90 ± 7.29%, p<0.05, Fig 4 and S2B Fig). Like IL-6 and IL-1β, the frequency of CD14^+^IL-8^+^ monocytes was decreased at presentation (52.31 ± 9.13% vs. 84.02 ± 2.68%, p<0.05, Fig 4 and S2C Fig), but was restored post-treatment (82.02 ± 3.64%, p<0.05, Fig 4 and S2C Fig). Contrary to expectation, the frequency of IL-12p40 significantly increased in PKDL *vis a vis* controls (2.37 ± 0.44% vs. 0.55 ± 0.11%, p<0.05, Fig 4 and S2D Fig). It remained elevated post-treatment (2.46 ± 0.38%, p<0.01, Fig 4 and S2D Fig), and was substantiated at the mRNA level (S3 Fig). The intramonocytic levels of TNF remained unchanged during active disease (2.49 ± 0.59% vs. 1.13 ± 0.36%) and with treatment (1.56 ± 0.40%).

A key anti-inflammatory cytokine secreted by M2 monocytes is TGF-β, a homodimer comprising LAP and TGF-β1. During cellular activation, cleavage of TGF-β1 from LAP results in TGF-β1 secretion. Therefore, the intramonocytic LAP-TGF-β1 complex indirectly reflects the status of TGF-β1 [22]. In PKDL, the frequency of LAP-TGF-β1 vs. controls was significantly reduced (4.26 ± 1.27% vs. 33.66 ± 10.44%, p<0.05 Fig 4 and S2E Fig), indicative of raised functional levels of TGF-β1, which increased with treatment (31.06 ± 8.65%, p<0.05, Fig 4 and S2E Fig). To confirm that this decrease in LAP-TGF-β1 was not attributable to decreased synthesis, mRNA expression of the *LAP-TGF-β1* complex was measured. A 10 fold increase was evident at presentation (31.05 ± 5.74 vs. 3.00 ± 0.44, p<0.05, S3 Fig) which decreased post-treatment (16.36 ± 4.84, S3 Fig).

As M2 polarization requires a milieu comprising IL-4, IL-10 and IL-13 [19], levels of these cytokines were estimated in patients with PKDL. The levels of IL-4 were significantly raised as compared to healthy individuals (126.80 ± 12.57 vs. 61.35 ± 6.36 pg/ml, p<0.05), as was IL-10 (37.06 ± 4.14 vs. 12.68 ± 2.05 pg/ml, p<0.01) and IL-13 (184.10 ± 45.22 vs. 7.77 ± 1.38 pg/ml, p<0.001), thus corroborating with previous reports [13]. Treatment caused a significant decrease in IL-10 (21.33 ± 3.66 pg/ml, p<0.01), reiterating its importance in leishmaniasis.

### Dermal macrophages showed M2 polarization

**Fig 5:**
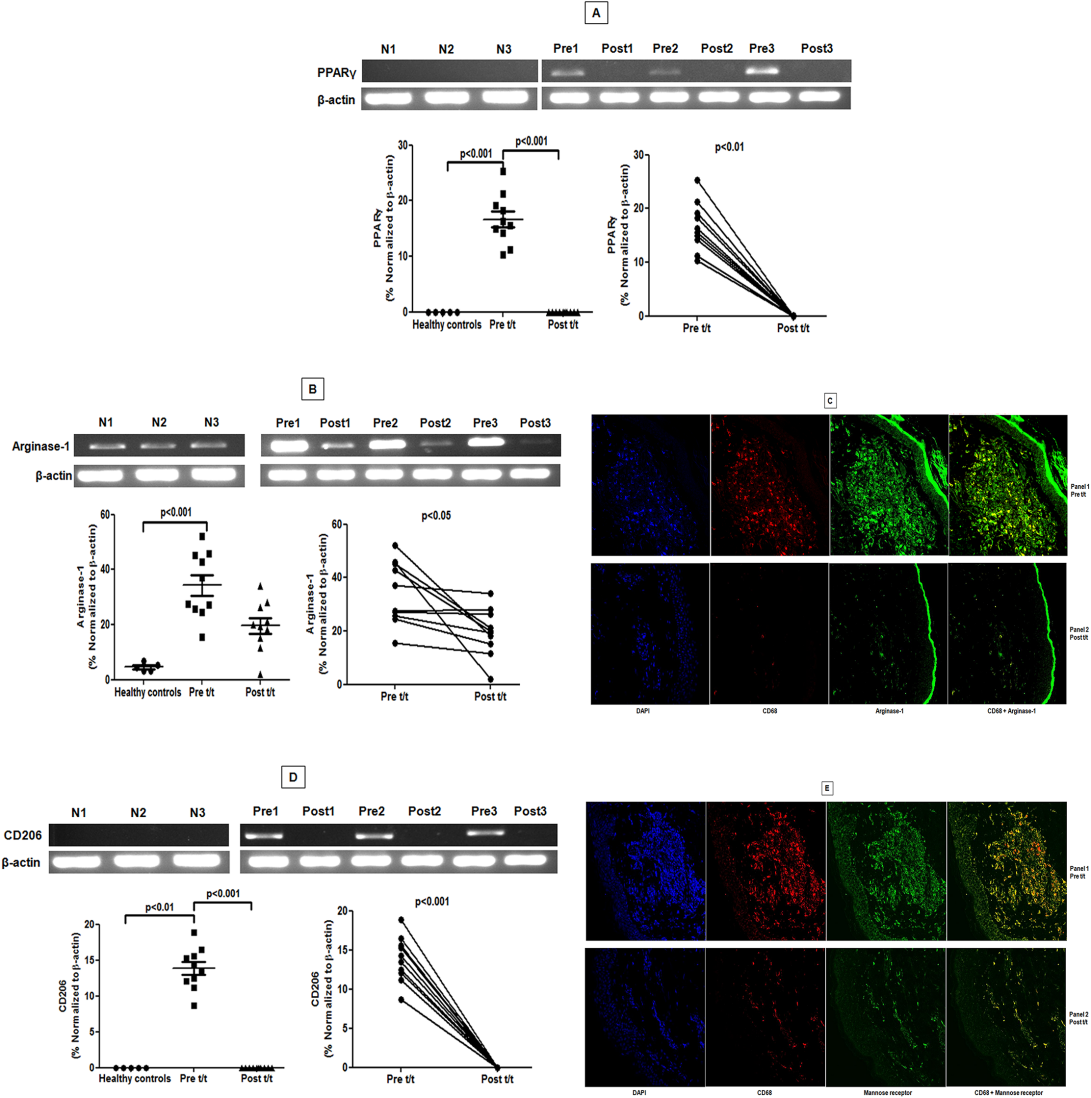
Lesional macrophages showed a raised expression of PPAR-γ, arginase-1 and mannose receptor. **A and B:** Representative mRNA expression profile of *PPARG* (A) and *ARG1* (B) in skin samples from healthy controls (N 1–3), patients with PKDL pre (Pre 1–3) and post treatment (Post 1–3). Scatter plots indicate RT-PCR products in healthy controls (●), patients with PKDL (Pre t/t, ■) and after treatment (Post t/t, ▲). These RT-PCR products were quantified by densitometric analysis after normalization with β-actin, along with before and after plots of the same. **C:** Expression of arginase-1 (green, Panels 1 and 2) in CD68^+^ macrophages (red, Panels 1 and 2) at the lesional site of a patient with PKDL (Pre t/t) and post treatment (Post t/t). Nuclei are shown in blue (DAPI, Panels 1 and 2). Co-localization of macrophage and arginase-1 appears as yellow. Figures were captured in 400X magnification. **D:** Representative mRNA expression profile of CD206 in dermal lesions of healthy controls (N 1–3), patients with PKDL pre (Pre 1–3) and post (Post 1–3) treatment. Scatter plots are as in A-B, above. **E:** Expression of mannose receptor (CD206, green, Panels 1 and 2) in CD68^+^ macrophages (red, Panels 1 and 2) at the lesional site of a patient with PKDL (Pre t/t) and post treatment (Post t/t). Nuclei are shown in blue (DAPI, Panels 1 and 2). Co-localization of CD68^+^ macrophages and mannose receptor (CD206) appears as yellow.

In patients with PKDL, an increased proportion of CD68^+^ macrophages was reported at the lesional sites, that regressed with treatment [23], and was corroborated in this study. An increased accumulation of *PPARγ* mRNA as compared to controls and post-treatment (Fig 5A) was accompanied by a significant increase in the mRNA of *ARG1* (Fig 5B). Confocal immunofluorescence confirmed localisation of arginase-1 within CD68^+^ macrophages. Post-treatment, the decrease in CD68^+^ was associated with a concomitant decrease in arginase-1 (Fig 5C).

The lesional 13.9 fold increase in *CD206* mRNA as compared to controls and post-treatment reinforced the M2 polarized status (13.86 ± 0.92; p<0.01 and p<0.001 respectively; Fig 5D). In addition, this was mirrored by raised protein expression evident via confocal microscopy (Fig 5E). With treatment, the decreased proportion of CD68^+^ macrophages resulted in a decreased expression of CD206 (Fig 5E). An expression of CD206 was also identified on CD68^-^ cells whose number also decreased with treatment, but their identity remains to be established. H&E staining confirmed the absence of polymorphonuclear cells (PMNs). Collectively, in PKDL, the M2 polarization that was evident in peripheral blood is also a consistent feature of monocytes/macrophages within dermal lesions.

### Vitamin-D signalling pathway during PKDL

**Fig 6:**
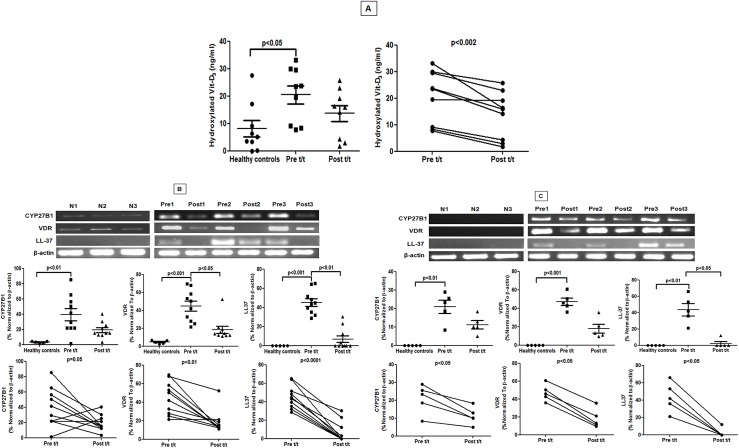
Status of Vitamin D3 and associated downstream signalling molecules in PKDL. **A:** Plasma levels of hydroxylated vitamin D3 in healthy controls (●) and patients with PKDL pre (Pre t/t, ■) and post treatment (Post t/t, ▲) along with before and after plots of the same. **B**: Representative mRNA expression profile of CYP27B1, VDR, LL-37 mRNA in circulating monocytes of healthy controls (N1-3), patients with PKDL (Pre1-3) and post treatment (Post1-3). Scatter plots indicate RT-PCR products in healthy controls (●), patients with PKDL (Pre t/t, ■) and after treatment (Post t/t, ▲). These RT-PCR products were quantified by densitometric analysis after normalization with β-actin, along with before and after plots of the same. **C:** Representative mRNA expression of CYP27B1, VDR, LL-37 in skin samples from healthy controls (N 1–3), patients with PKDL (Pre 1–3) and post treatment (Post 1–3). Scatter plot indicates expression as in B above.

As Vitamin D receptor signaling has been linked to M2 polarization and generation of antimicrobial peptides [24], this pathway was examined as it may underlie the systemic and local M2 polarization in PKDL. Plasma 1α,25-dihydroxyvitamin D_3_(1,25D3) was significantly raised during PKDL, compared to controls and post-treatment (20.50 ± 3.32 vs. 8.05 ± 2.99 vs. 13.68 ± 2.92 ng/mL respectively, Fig 6A). The bioactive 1,25D3 is generated from its inactive prohormone by vitamin D-1α-hydroxylase, encoded by *CYP27B1*. In keeping with the elevated plasma levels of 1,25D3, an increase in *CYP27B1* mRNA accumulation was evident (Fig 6B and 6C). VDR is responsible for nuclear signaling of 1,25D3, and its accumulation in patient monocytes and skin biopsies was accompanied with an 11-fold increase in the downstream antimicrobial effector peptide cathelecidin (hCAP18/LL-37, Fig 6B and 6C). Treatment reduced most components of the Vitamin D signalling pathway, though levels did not always return to levels comparable with controls (Fig 6B and 6C).

## Discussion

Papulo-nodules in PKDL being parasite-rich have fuelled speculation of its pivotal role in the transmission of VL especially in South Asia, where VL is anthroponotic making patients with PKDL the strongest contenders to be the disease reservoir. Accordingly, its eradication should be an essential component of the VL elimination programme [http://www.who.int/tdr/publications/documents/kala_azar_indicators.pdf; last accessed on 1^st^ March 2015], emphasising the importance of establishing a greater understanding of the immunopathogenesis of PKDL. In the absence of an experimental model, the challenge of delineating the immunoclinical determinants of PKDL lies squarely on the shoulders of clinical researchers which constituted the focus of this study (Fig 1).

In experimental and human VL, attenuation of the oxidative burst, secondary to reduced phosphorylation of MAPKs occurred through the TLR-2 pathway [25, 26] or the CD40 signalosome [27]. Similarly in PKDL, the decreased expression of TLR-2/4 might also translate into impaired MAPK signalling, resulting in the intramonocytic redox imbalance tilting towards an anti-inflammatory milieu (Fig 2). Importantly, with chemotherapy, the generation of NO, but not ROS was significantly increased (Fig 2). This was concordant with studies in VL wherein NO played a pivotal role and was associated with TLR-4 [26,15]. However, in patients with CL (*L*. *braziliensis* and/or *L*. *guyanenesis)*, NO played an insignificant role, ROS being more important [28], suggesting that alterations in the redox status are attributable to parasite species and disease manifestations. Taken together, monocyte-macrophage dysfunction is a hallmark of PKDL and is facilitated by the immunosuppressive microenvironment of IL-4, IL-13 and IL-10 [13], which are essential stimuli for driving monocytes towards innate deactivation or alternate activation.

In mouse monocytes/macrophages, the intricate network of signalling molecules, associated transcription factors along with post transcriptional regulators mediating the different forms of activation are well delineated [29]. IL-4 and IL-13 via STAT6 activation are known to skew the macrophage function towards the M2 phenotype leading to transcription of genes typical of M2 polarization, notably Mannose receptor (*Mrc1*), Arginase (*Arg1*), PPARγ (*PPARG*) and *Fizz1* among others [12]. However, efforts to simulate the murine scenario by pulsing human monocyte derived macrophages with IL-4 or IL-13 failed to demonstrate arginase-1 expression, based on which conclusions were inferred that *Arg1* is strictly restricted to mouse M2 cells [11]. However, this *ex-vivo* situation may well be a cytokine induced artefact, as *in vivo*, human monocytes require multiple signals to establish the entire spectrum of alternative activation [30]. Indeed, in patients with filariasis, an increased expression of arginase-1 in M2 monocytes was demonstrated [31]. This was mirrored in our study and endorsed our proposition that in parasitic diseases, an enhanced expression of arginase-1 is a feature of M2 polarized human monocytes/macrophages (Fig 3B). Studies in human VL and CL have also demonstrated increased arginase activity sourced from low density granulocytes [8,9]. Importantly, this increased presence of arginase supports parasite survival by increasing the availability of polyamines [10, 32]. Additionally, the decreased availability of the microbicidal nitric oxide would also support disease progression. In a hamster model of VL, *L*. *donovani* directly induced STAT-6 phosphorylation and increased arginase-1 leading to disease progression [33, 34]. The process was augmented by exogenous IL-4 and other factors like insulin like growth factor 1(IGF-1) and fibroblast growth factor [FGF, 33, 34]. Furthermore, the raised plasma arginase [13] translated into depletion of L-arginine and led to an impairment of T cell functions [32]. Taken together, induction of arginase-1 drives disease progression and its blockade might support disease resolution.

Although during PKDL, monocytes/macrophages parasites reside primarily in dermal lesions, changes were observed in systemic monocytes (Figs 3 and 4) suggesting that M2 polarization is not a direct consequence of intracellular subversion strategies employed by the parasite [35]. However, we cannot formally rule out the possibility that parasite-derived immune modulators e.g. in exosomes [36] mediated this effect. As plasma IL-4, IL-10, IL-13 and TGF-β was elevated in PKDL [13], it is more likely that polarization occured following bystander responses to inflammation and immune activation [37]. As monocytes are yet to be segregated into M1 and M2, it would be prudent to endorse the increased expression of PPARγ, Arg1 and CD206 (Fig 3A–3D) by complimentary studies wherein biomarker expression of infected monocytes should be compared with uninfected monocytes after being pulsed with IL-4 and/or IL-13.

A key factor for development of the M2 phenotype is activation of PPARγ, a transcription factor of the nuclear hormone receptor family that acts downstream of STAT6 signalling to regulate macrophage metabolism. In experimental models of leishmaniasis [38], upregulation of PPARγ by IL-4 was demonstrated to instill monocytes/macrophages with potent anti-inflammatory and Th2 functionalities [38], essential for disease progression. PPARγ through its transrepressive action blocks expression of iNOS and NF-*κ*B mediated transcription of pro-inflammatory mediators [39]. In addition, cytosolic PPARγ by interfering with activation of PKC-α can suppress NADPH oxidase and impair generation of superoxide and NO. Furthermore, as PPARγ is responsible for the enhanced expression of CD206-mannose receptor [40], their increased expression at disease presentation in lesional macrophages and circulating monocytes, provided strong endorsement of M2 monocyte/macrophage polarization (Figs 3 and 5). The presence of CD68^-^CD206^+^ cells in dermal lesions could be inflammatory dermal dendritic cells, which although absent in normal human skin have been demonstrated in the epidermis of patients with psoriasis and atopic dermatitis [41,42]. Ideally, to specifically address the relevance of CD68^+^ macrophages in disease outcome, the features of M2 polarization should be demonstrated in isolated CD68^+^ macrophages by comparing arginase expression in macrophages vs. arginase expressed by other cells e.g. low density neutrophils. However, owing to limited availability of clinical material, this was not feasible and importantly, PMNs were absent in H&E stained sections at disease presentation and post treatment.

The M1 monocytes/macrophages produce primarily pro-inflammatory cytokines (IL-6, IL-8, IL-1β, TNF-α and IL-12), whereas M2 monocytes sustain their immunoregulatory and immunosuppressive phenotype via IL-10 and TGF-β [4]. In agreement, monocytes in PKDL patients generated lower amounts of IL-6, IL-1β and IL-8 (Fig 4 and S2A–S2C Fig). Interestingly, a small but significant population of monocytes expressed the pro-inflammatory IL-12p40, and their frequency remained high post treatment (Fig 4 and S2D Fig), similar to a study by Gupta *et al* [43]. In PKDL, IL-10 and TGF-β, signature cytokines of M2 monocytes play a vital role in disease progression [44]^.^ This was confirmed by our study wherein circulating monocytes were established to be a rich source of TGF-β (Fig 4E). With treatment, the decrease of TGF-β in monocytes strengthened the notion that in PKDL, monocytes are alternatively activated, with treatment repolarizing them towards a M1 phenotype.

As lesions in PKDL tend to mirror clothing habits and consistently appear in sun exposed areas [1,2], the contribution of Vitamin D_3_, whose synthesis increases following UV or sunlight exposure was explored. VitD_3_ is a potent immunosuppressant of macrophages/monocytes that downregulates TLR-2/4 and monocyte co-stimulatory molecules [45]. Additionally, VitD_3_ inhibits production of intramonocytic pro-inflammatory cytokines by modulating the MAPK phosphatase-1 [46] and induces M2 polarization [47]. In Behcet’s disease, a chronic inflammatory disorder, TLR 2/4 expression negatively correlated with vitamin D_3_ and importantly, the dose dependent treatment of vitamin D_3_ decreased inflammation, as also decreased the expression of TLR-2/4 [48]. Our data in PKDL suggests a similar scenario as decreased expression of TLR 2/4 was concomitant with increased vitamin D_3_ (Figs 2 and 6). This was in concordance with a *L*. *major* model wherein VDR knockout mice showed resistance to infection. Alongside, addition of 1,25(OH)_2_D_3_ to *L*. *major*-infected macrophages translated into induction of arginase-1, down regulation of iNOS and parasite persistence [49]. Similarly, in PKDL, raised serum 25(OH)D_3_ was accompanied by an enhanced mRNA expression of CYP27B1, VDR and LL-37, indicating that infection upregulated the molecular switch needed for monocyte polarization towards a M2 phenotype (Fig 6).

Differential polarization of macrophages in diverse disease conditions confirms the plasticity of macrophages, with M1 polarization evident in inflammatory and autoimmune diseases such as diabetes, atherosclerosis and sepsis, while a strong M2 or M2-like polarization has been proposed in cancers, chronic parasitic, viral or bacterial diseases [4,19]. Conversely, the inability to switch to an M2 phenotype may underlie the failure to resolve inflammation e.g. chronic venous ulcers [50]. Like in tumors, M2 polarized macrophages and dendritic cells, have been proposed to contribute towards subversion of adaptive immunity thus promoting tumor growth and progression [51], it may be envisaged that the *Leishmania* parasite ensures its survival by creating an immunosuppressive milieu via M2 polarization of macrophages and a decrease in dendritic cells [23], thus collectively causing impairment of antigen presentation. Ideally, studies confirming the functional phenotype are recommended and best addressed in an *ex vivo* assay, the limiting factor being the availability of lesional material. Accordingly, reorientation of these polarized macrophages is now an integral component of macrophage targeted therapy [19]. It can be envisaged that in diseases like leishmaniasis, the strategy of reshaping and reorientation of macrophage polarization could be a promising therapeutic modality worthy of future consideration.

## Supporting Information

S1 Table List of primers. (DOC)

S1 Fig Analysis of monocyte subsets in patients with PKDL. Representative quadrant plot showing frequency of monocyte subsets, classical (CD14^++^16^-^) intermediate (CD14^++^16^+^) and non-classical (CD14^+^16^+^) in a healthy control, a patient with PKDL (Pre t/t) and post treatment (Post t/t). Monocytes were initially gated on the basis of their morphology (forward vs. side scatter) and then classified based on their CD14 and CD16 positivity. R2 represents classical (CD14^++^16^-^), R3 represents intermediate (CD14^++^16^+^) and R4 represents non-classical (CD14^+^16^+^) monocytes. Frequency of monocyte subsets, namely CD14^++^16^-^ (blank square), CD14^++^16^+^ (black square) and CD14^+^16^+^ (diagonally lined square) in healthy controls, patients with PKDL (Pre t/t) and after treatment (Post t/t).
(TIF)

S2 Fig Intramonocytic status of cytokines in PKDL. **A-E:** Representative data showing expression of IL-6 (**A**), IL-1β (**B**), IL-8 (**C**), IL-12p40 (**D**) and LAP-TGF-β1 (**E**) in CD14^+^ monocytes from a healthy control, patient with PKDL (Pre t/t) and after treatment (Post t/t).
(TIF)

S3 Fig mRNA expression of *IL-12p40* and *LAP-TGF-β1* in patients with PKDL. Representative dermal mRNA expression profiles of *IL-12p40* and *β-actin* in blood samples from healthy controls (N 1–3), patients with PKDL (Pre 1–3) and after treatment (Post 1–3). Scatter plots indicate RT-PCR products in healthy controls (●), patients with PKDL (Pre t/t, ■) and after treatment (Post t/t, ▲). These RT-PCR products were quantified by densitometric analysis after normalization with β-actin, along with before and after plots of the same.
(TIF)

## References

[1] Mukhopadhyay D, Dalton JE, Kaye PM, Chatterjee M. Post kala-azar dermal leishmaniasis: an unresolved mystery. Trends Parasitol. 2014;30: 65–74. 10.1016/j.pt.2013.12.004 24388776 PMC3919212

[2] Zijlstra EE, Musa AM, Khalil EA, el-Hassan IM, el-Hassan AM. Post kala-azar dermal leishmaniasis. Lancet Infect Dis. 2003;3: 87–98. 12560194 10.1016/s1473-3099(03)00517-6

[3] Mantovani A, Biswas SK, Galdiero MR, Sica A, Locati M. Macrophage plasticity and polarization in tissue repair and remodelling. J Pathol. 2013;229: 176–185. 10.1002/path.4133 23096265

[4] Martinez FO, Helming L, Gordon S. Alternative activation of macrophages: an immunologic functional perspective. Annu Rev Immunol. 2009;27: 451–483. 10.1146/annurev.immunol.021908.132532 19105661

[5] Pearce EJ, MacDonald AS. The immunobiology of schistosomiasis. Nat Rev Immunol. 2002;2: 499–511. 12094224 10.1038/nri843

[6] Biswas SK, Chittezhath M, Shalova IN, Lim JY. Macrophage polarization and plasticity in health and disease. Immunol Res. 2012;53: 11–24. 10.1007/s12026-012-8291-9 22418728

[7] Hölscher C, Arendse B, Schwegmann A, Myburgh E, Brombacher F. Impairment of alternative macrophage activation delays cutaneous leishmaniasis in nonhealing BALB/c mice. J Immunol. 2006;176: 1115–1121. 16394000 10.4049/jimmunol.176.2.1115

[8] Abebe T, Hailu A, Woldeyes M, Mekonen W, Bilcha K, Cloke T et al Local increase of arginase activity in lesions of patients with cutaneous leishmaniasis in Ethiopia. PLoS Negl Trop Dis. 2012; 6: e1684 10.1371/journal.pntd.0001684 22720104 PMC3373636

[9] Abebe T, Takele Y, Weldegebreal T, Cloke T, Closs E, Corset C et al Arginase activity—a marker of disease status in patients with visceral leishmaniasis in Ethiopia. PLoS Negl Trop Dis. 2013;7: e2134 10.1371/journal.pntd.0002134 23556019 PMC3610885

[10] França-Costa J, Van Weyenbergh J, Boaventura VS, Luz NF, Malta-Santos H, Oliveira MC et al Arginase I, polyamine, and prostaglandin E2 pathways suppress the inflammatory response and contribute to diffuse cutaneous leishmaniasis. J Infect Dis. 2015;211: 426–435. 10.1093/infdis/jiu455 25124926

[11] Raes G, Van den Bergh R, De Baetselier P, Ghassabeh GH, Scotton C, Locati M, et al Arginase-1 and Ym1 are markers for murine, but not human, alternatively activated myeloid cells. J Immunol. 2005;174: 6561 15905489 10.4049/jimmunol.174.11.6561

[12] Martinez FO, Helming L, Milde R, Varin A, Melgert BN, Draijer C, et al Genetic programs expressed in resting and IL-4 alternatively activated mouse and human macrophages: similarities and differences. Blood. 2013;121: e57–69. 10.1182/blood-2012-06-436212 23293084

[13] Mukhopadhyay D, Das NK, Roy S, Kundu S, Barbhuiya JN, Chatterjee M. Miltefosine effectively modulates the cytokine milieu in Indian post kala-azar dermal leishmaniasis. J Infect Dis. 2011;204: 1427–1436. 10.1093/infdis/jir551 21933878

[14] Sarkar A, Saha P, Mandal G, Mukhopadhyay D, Roy S, Singh S.K, et al Monitoring of intracellular nitric oxide in leishmaniasis: its applicability in patients with visceral leishmaniasis. Cytometry A. 2011;79: 35–45. 10.1002/cyto.a.21001 21182181

[15] Sarkar A, Mandal G, Singh N, Sundar S, Chatterjee M. Flow cytometric determination of intracellular non-protein thiols in *Leishmania* promastigotes using 5-chloromethyl fluorescein diacetate. Exp Parasitol. 2009;122: 299–305. 10.1016/j.exppara.2009.04.012 19393240

[16] Kundu S, Bala A, Ghosh P, Mukhopadhyay D, Mitra A, Sarkar A, et al Attenuation of oxidative stress by allylpyrocatechol in synovial cellular infiltrate of patients with Rheumatoid Arthritis. Free Radic Res. 2011;45: 518–526. 10.3109/10715762.2011.555480 21284489

[17] Ziegler-Heitbrock L, Ancuta P, Crowe S, Dalod M, Grau V, Hart DN et al Nomenclature of monocytes and dendritic cells in blood. Blood. 2010;116: e74–80. 10.1182/blood-2010-02-258558 20628149

[18] Gordon S. Alternative activation of macrophages. Nat Rev Immunol. 2003;3: 23–35. 12511873 10.1038/nri978

[19] Sica A, Mantovani A. Macrophage plasticity and polarization: *in vivo veritas* . J Clin Invest. 2012;122: 787–795. 10.1172/JCI59643 22378047 PMC3287223

[20] Chawla A. Control of macrophage activation and function by PPARs. *Circ*. *Res*. 2010;106: 1559–1569. 20508200 10.1161/CIRCRESAHA.110.216523 PMC2897247

[21] Ansari NA, Ramesh V, Salotra P. Interferon (IFN)-gamma, tumor necrosis factor-alpha, interleukin-6, and IFN-gamma receptor 1 are the major immunological determinants associated with post-kala azar dermal leishmaniasis. J Infect Dis. 2006;194: 958–965. 16960784 10.1086/506624

[22] Yoshinaga K, Obata H, Jurukovski V, Mazzieri R, Chen Y, Zilberberg L, et al Perturbation of transforming growth factor (TGF)-beta1 association with latent TGF-beta binding protein yields inflammation and tumors. Proc Natl Acad Sci U S A. 2009;105: 18758–18763. 10.1073/pnas.0805411105 PMC2596235 19022904

[23] Mukherjee S, Mukhopadhyay D, Braun C, Barbhuiya JN, Das NK, Chatterjee U, et al Decreased presence of Langerhans cells are critical determinants for Indian Post kala-azar dermal leishmaniasis. Exp Dermatol. 2015;24: 232–234. 10.1111/exd.12635 25580856

[24] Griffin MD, Xing N, Kumar R. Vitamin D and its analogs as regulators of immune activation and antigen presentation. Annu Rev Nutr. 2003;23: 117–145. 12651965 10.1146/annurev.nutr.23.011702.073114

[25] Bhattacharya P, Gupta G, Majumder S, Adhikari A, Banerjee A, Halder K, et al Arabinosylated lipoarabinomannan skews Th2 phenotype towards Th1 during *Leishmania* infection by chromatin modification: involvement of MAPK signaling. PLoS One. 2011;6: e24141 10.1371/journal.pone.0024141 21935379 PMC3173371

[26] Roy S, Mukhopadhyay D, Mukherjee S, Ghosh S, Kumar S, Sarkar K, et al A defective oxidative burst and impaired antigen presentation are hallmarks of human Visceral Leishmaniasis. J Clin Immunol. 2015;35: 56–67. 25479930 10.1007/s10875-014-0115-3

[27] Rub A, Dey R, Jadhav M, Kamat R, Chakkaramakkil S, Majumdar S, et al Cholesterol depletion associated with *Leishmania major* infection alters macrophage CD40 signalosome composition and effector function. Nat. Immunol. 2009;10: 273–280. 10.1038/ni.1705 19198591

[28] Novais FO, Nguyen BT, Beiting DP, Carvalho LP, Glennie ND, Passos S, et al Human classical monocytes control the intracellular stage of *Leishmania braziliensis* by reactive oxygen species. J Infect Dis. 2014;209: 1288–1296. 10.1093/infdis/jiu013 24403561 PMC3969552

[29] Mantovani A, Sica A, Sozzani S, Allavena P, Vecchi A, Locati M. The chemokine system in diverse forms of macrophage activation and polarization. Trends Immunol. 2004;25: 677–686. 15530839 10.1016/j.it.2004.09.015

[30] Scott AL. The alternatively activated human-redux. J Infect Dis. 2009;199: 1723–1725. 10.1086/599091 19456228

[31] Babu S, Kumaraswami V, Nutman TB. Alternatively activated and immunoregulatory monocytes in human filarial infections. J Infect Dis. 2009;199: 1827–1837. 10.1086/599090 19456233 PMC3440875

[32] Stempin CC, Dulgerian LR, Garrido VV, Cerban FM. Arginase in parasitic infections: macrophage activation, immunosuppression, and intracellular signals. J Biomed Biotechnol. 2010;2010: 683485 10.1155/2010/683485 20029630 PMC2792949

[33] Osorio EY, Zhao W, Espitia C, Saldarriaga O, Hawel L, Byus CV et al Progressive visceral leishmaniasis is driven by dominant parasite-induced STAT6 activation and STAT6-dependent host arginase 1 expression. PLoS Pathog. 2012;8: e1002417 10.1371/journal.ppat.1002417 22275864 PMC3261917

[34] Osorio EY, Travi BL, da Cruz AM, Saldarriaga OA, Medina AA, Melby PC. Growth factor and Th2 cytokine signaling pathways converge at STAT6 to promote arginase expression in progressive experimental visceral leishmaniasis. PLoS Pathog. 2014;10: e1004165 10.1371/journal.ppat.1004165 24967908 PMC4072777

[35] Kaye P, Scott P. Leishmaniasis: complexity at the host-pathogen interface. Nat Rev Microbiol. 2011;9: 604–615. 10.1038/nrmicro2608 21747391

[36] Lambertz U, Silverman JM, Nandan D, McMaster WR, Clos J, Foster LJ, et al Secreted virulence factors and immune evasion in visceral leishmaniasis. J Leukoc Biol. 2012;91: 887–899. 10.1189/jlb.0611326 22442494

[37] Beattie L, d'El-Rei Hermida M, Moore JW, Maroof A, Brown N, Lagos D, et al A transcriptomic network identified in uninfected macrophages responding to inflammation controls intracellular pathogen survival. Cell Host Microbe. 2013;14: 357–368. 10.1016/j.chom.2013.08.004 24034621 PMC4180915

[38] Chan MM, Adapala N and Chen C. Peroxisome Proliferator-Activated Receptor-γ-mediated polarization of macrophages in Leishmania infection. PPAR Res. 2012;2012: 796235 10.1155/2012/796235 22448168 PMC3289877

[39] Brüne B, Dehne N, Grossmann N, Jung M, Namgaladze D, Schmid T, et al Redox control of inflammation in macrophages. Antioxid Redox Signal. 2013;19: 595–637. 10.1089/ars.2012.4785 23311665 PMC3718318

[40] Bouhlel MA, Derudas B, Rigamonti E, Dièvart R, Brozek J, Haulon S, et al PPARgamma activation primes human monocytes into alternative M2 macrophages with anti-inflammatory properties. Cell Metab. 2007;6: 137–143. 17681149 10.1016/j.cmet.2007.06.010

[41] Wollenberg A, Mommaas M, Oppel T, Schottdorf EM, Günther S and Moderer M. Expression and function of the mannose receptor CD206 on epidermal dendritic cells in inflammatory skin diseases. J Invest Dermatol. 2002;118: 327–334. 11841552 10.1046/j.0022-202x.2001.01665.x

[42] Angel CE, Lala A, Chen CJ, Edgar SG, Ostrovsky LL, Dunbar PR. CD14^+^ antigen-presenting cells in human dermis are less mature than their CD1a^+^ counterparts. Int Immunol. 2007;19: 1271–1279. 17804688 10.1093/intimm/dxm096

[43] Gupta R, Kushawaha PK, Samant M, Jaiswal AK, Baharia RK and Dube A. Treatment of *Leishmania donovani*-infected hamsters with miltefosine: analysis of cytokine mRNA expression by real-time PCR, lymphoproliferation, nitrite production and antibody responses. J Antimicrob Chemother. 2012;67: 440–443. 10.1093/jac/dkr485 22121191

[44] Saha S, Mondal S, Ravindran R, Bhowmick S, Modak D, Mallick S, et al IL-10- and TGF-beta-mediated susceptibility in kala-azar and post-kala-azar dermal leishmaniasis: the significance of amphotericin B in the control of *Leishmania donovani* infection in India. J Immunol. 2007;179: 5592–5603. 17911647 10.4049/jimmunol.179.8.5592

[45] Sadeghi K, Wessner B, Laggner U, Ploder M, Tamandl D, Friedl J, et al Vitamin D3 down-regulates monocyte TLR expression and triggers hyporesponsiveness to pathogen-associated molecular patterns. Eur J Immunol. 2006;36: 361–370. 16402404 10.1002/eji.200425995

[46] Zhang Y, Leung DY, Richers BN, Liu Y, Remigio LK, Riches DW, et al. Vitamin D inhibits monocyte/macrophage proinflammatory cytokine production by targeting MAPK phosphatase-1. J Immunol. 2012;188: 2127–2135. 10.4049/jimmunol.1102412 22301548 PMC3368346

[47] Mantovan A, Sica A and Locati M. Macrophage polarization comes of age. Immunity. 2005;23: 344–346. 16226499 10.1016/j.immuni.2005.10.001

[48] Do JE, Kwon SY, Park S and Lee ES. Effects of vitamin D on expression of Toll-like receptors of monocytes from patients with Behcet's disease. Rheumatology. 2008;47: 840–848. 10.1093/rheumatology/ken109 18411217

[49] Ehrchen J, Helming L, Varga G, Pasche B, Loser K, Gunzer M, et al. Vitamin D receptor signalling contributes to susceptibility to infection with *Leishmania major* . FASEB J. 2007;21: 3208–3218. 17551101 10.1096/fj.06-7261com

[50] Sindrilaru A, Peters T, Wieschalka S, Baican C, Baican A, Peter H, et al An unrestrained proinflammatory M1 macrophage population induced by iron impairs wound healing in humans and mice. J Clin Invest. 2011;121: 985–997. 10.1172/JCI44490 21317534 PMC3049372

[51] Mantovani A, Sozzani S, Locati M, Allavena P, Sica A. Macrophage polarization: tumor-associated macrophages as a paradigm for polarized M2 mononuclear phagocytes. Trends Immunol. 2002;23: 549–55. 12401408 10.1016/s1471-4906(02)02302-5

