# Supplementary material for: M2 Polarization of Monocytes-Macrophages Is a Hallmark of Indian Post Kala-Azar Dermal Leishmaniasis
Source: PLoS Negl Trop Dis. 2015 Oct 23;9(10):e0004145. doi: 10.1371/journal.pntd.0004145 (PMC4619837; doi:10.1371/journal.pntd.0004145)
Supplement: S1 Table — (DOC) [file pntd.0004145.s001.doc]

Supplementary Table 1: Primer details

| **Name** | **Sequence (5’ 3’)** | **Annealing temperature (^o^ C)** | **Product Size (bp)** |
| --- | --- | --- | --- |
| IL-12p40 (F) | GCCGTTCACAAGCTCAAGTATG | 66 | 79 |
| IL-12p40 (R) | TCTTGGGTGGGTCAGGTTTG |  |  |
| LAP-TGF-β1 (F) | GTACCTGAACCCGTGTTGCT | 68 | 405 |
| LAP-TGF-β1 (R) | TACAGCTGCCGCACGCAGCA |  |  |
| Arginase-1 (F) | ACTGGAGAGCTCAAGTGCAGCAA | 61 | 308 |
| Arginase-1 (R) | AGCCTTGGCTGAGATCACGAGCC |
| Mannose receptor (F) | GGCGGTCGGATGGATGGCTC | 62 | 610 |
| Mannose receptor (R) | TGTGAGGTCACCGCCTTCCT |
| PPAR-γ (F) | TCTGGCCCACCAACTTTGGG | 59 | 340 |
| PPAR-γ (R) | CTTCACAAGCATGAACTCCA |
| CYP27B1 (F) | GGTCAGGGCCGCCTCACACT | 60 | 396 |
| CYP27B1 (R) | GGCCACAGGTGCCACCCAATC |
| Vitamin D Receptor (F) | CCAGTTCGTGTGAATGATGG | 55 | 384 |
| Vitamin D Receptor (R) | GTCGTCCATGGTGAAGGACT |
| LL-37 (F) | GGACCCAGACACGCCAAA | 60 | 51 |
| LL-37 (R) | GCACACTGTCTCCTTCACTGTGA |
| β-actin (F) | CCCAAGGCCAACCGCGAGAAGAT | 74 | 224 |
| β-actin (R) | GTCCCGGCCAGCCAGGTCCAG |  |  |
