# Supplementary figures and images for: M2 Polarization of Monocytes-Macrophages Is a Hallmark of Indian Post Kala-Azar Dermal Leishmaniasis
Source: PLoS Negl Trop Dis. 2015 Oct 23;9(10):e0004145. doi: 10.1371/journal.pntd.0004145 (PMC4619837; doi:10.1371/journal.pntd.0004145)

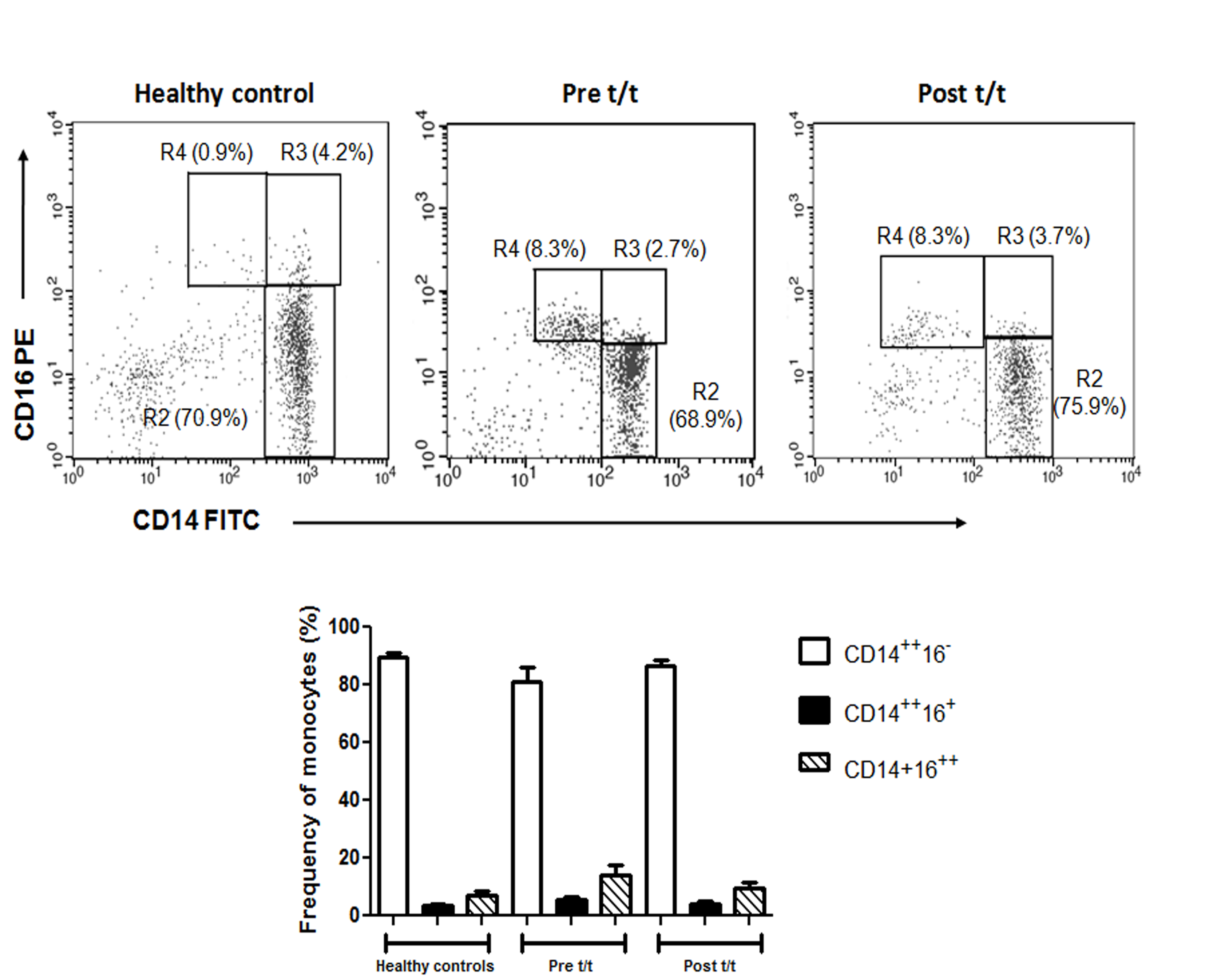

Supplement: S1 Fig — Representative quadrant plot showing frequency of monocyte subsets, classical (CD14++16-) intermediate (CD14++16+) and non-classical (CD14+16+) in a healthy control, a patient with PKDL (Pre t/t) and post treatment (Post t/t). Monocytes were initially gated on the basis of their morphology (forward vs. side scatter) and then classified based on their CD14 and CD16 positivity. R2 represents classical (CD14++16-), R3 represents intermediate (CD14++16+) and R4 represents non-classical (CD14+16+) monocytes. Frequency of monocyte subsets, namely CD14++16- (blank square), CD14++16+ (black square) and CD14+16+ (diagonally lined square) in healthy controls, patients with PKDL (Pre t/t) and after treatment (Post t/t). (TIF) [file pntd.0004145.s002.tif]

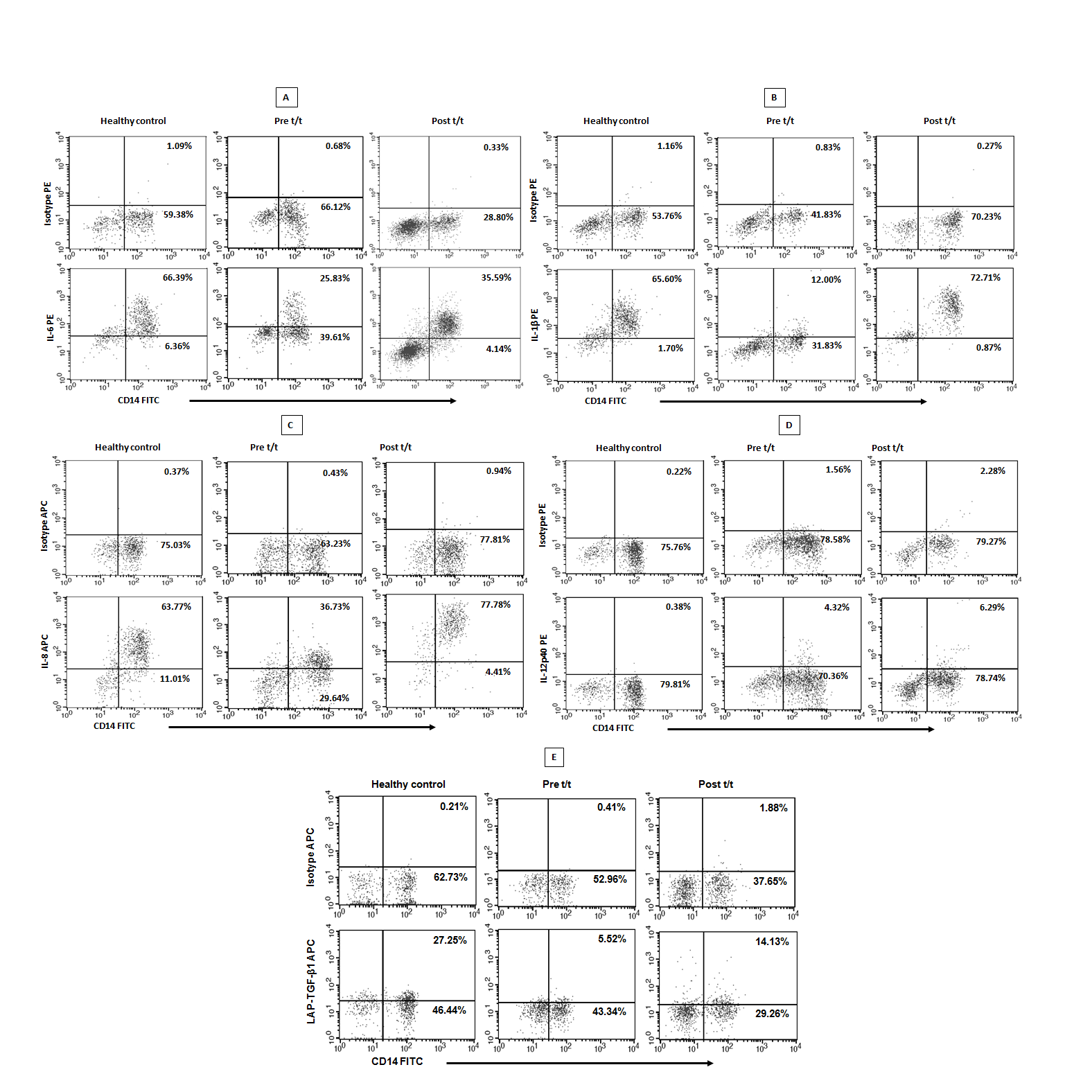

Supplement: S2 Fig — A-E: Representative data showing expression of IL-6 (A), IL-1β (B), IL-8 (C), IL-12p40 (D) and LAP-TGF-β1 (E) in CD14+ monocytes from a healthy control, patient with PKDL (Pre t/t) and after treatment (Post t/t). (TIF) [file pntd.0004145.s003.tif]

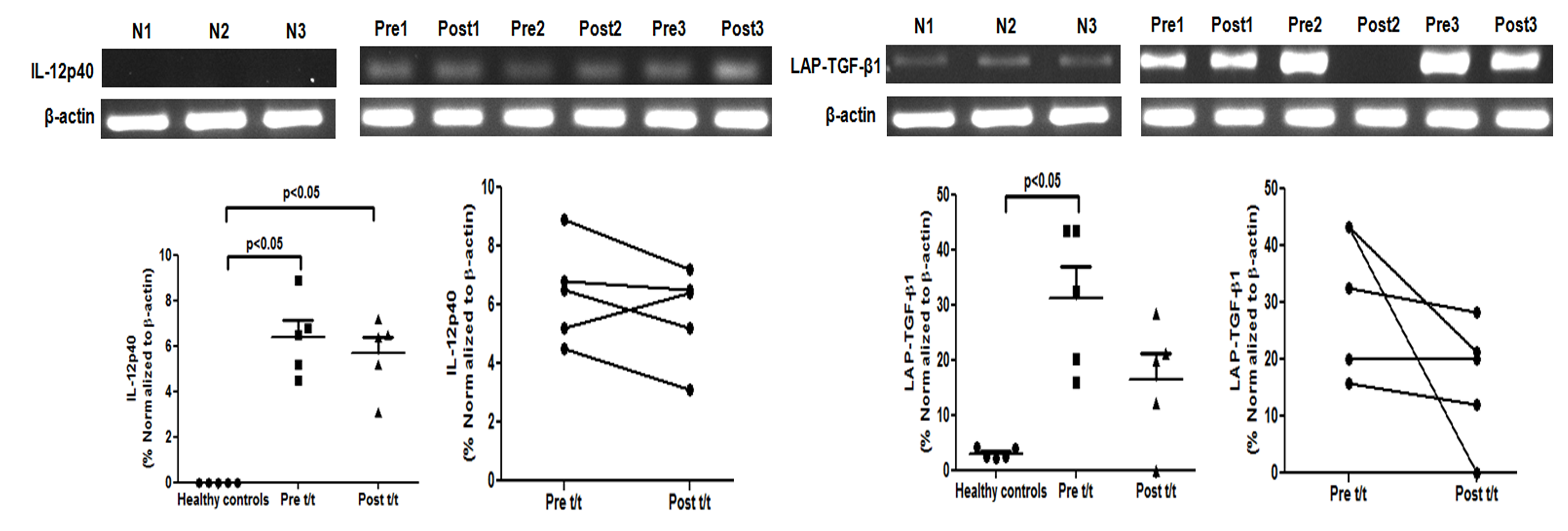

Supplement: S3 Fig — Representative dermal mRNA expression profiles of IL-12p40 and β-actin in blood samples from healthy controls (N 1–3), patients with PKDL (Pre 1–3) and after treatment (Post 1–3). Scatter plots indicate RT-PCR products in healthy controls (●), patients with PKDL (Pre t/t, ■) and after treatment (Post t/t, ▲). These RT-PCR products were quantified by densitometric analysis after normalization with β-actin, along with before and after plots of the same. (TIF) [file pntd.0004145.s004.tif]
